# Hospital Meetings

**Published:** 1904-05-21

**Authors:** 


### HOSPITAL MEETINGS.

### BRITISH HOME AND HOSPITAL FOR INCURABLES.

The annual general meeting of the governors and subscribers of this institution was held on May 12th, under the presidency of Mr. J. H. Hale, deputy-chairman of the board of management, in the unavoidable absence of Lord Amherst.

The Chairman congratulated his hearers on the satisfactory nature of the report. The net ordinary income for the year, exclusive of legacies or capital donations was £8,175, while the expenditure was £15,191, and in order to meet this the board had been compelled to utilise upwards of £6,000 from legacies for current expenses, instead of treating them all as capital. The total of annual subscriptions was £3,107, whereas the sum paid in pensions alone amounted to £6,959. The board felt that the subscriptions ought to realise sufficient to pay for the pensions, and he appealed earnestly to all present to try and each get one new subscriber for the coming year. The capital gifts, though warmly welcomed by the Board, did not lessen the expenses of the Home, but on the other hand left a considerable margin to be made up out of the funds. After referring to the law action that had recently taken place between the institution and the Royal Hospital for Incurables, Putney, over a legacy of £500 that had been left by an old subscriber of the Streatham Home and which had been awarded to the Putney Hospital, Mr. Hale expressed the hope that the amount thus lost would be made up by increased subscriptions.

The Queen had again shown her interest in the institution by sending the sum of £12 18s., being a further portion of the proceeds of the sale of Canon Fleming's sermon. He had also recently received a letter from the Queen's secretary in which he said that though her Majesty could not settle the day on which she would visit the Home till after Whitsuntide, she was certainly coming and was looking forward to doing so.

The loan from the bankers of £1,000 was still unpaid, and at the close of the financial year there was a sum of £785 due to tradesmen and others.

He was glad to be able to announce that the whole cost of installing electric light in the Home had been defrayed by the kindness of a friend.

The report having been adopted, the list of successful candidates was read and other business transacted.

